# Revealing the Potential Associations of Mutation-Related Genes with Lymph Node Metastasis in Gallbladder Cancer Through Transcriptome and Exome Sequencing

**DOI:** 10.3390/biomedicines14051076

**Published:** 2026-05-10

**Authors:** Qi Li, Qingyu Tang, Dong Xue, Hengchao Liu, Zhenqi Tang, Dong Zhang, Chen Chen, Zhimin Geng

**Affiliations:** Department of Hepatobiliary Surgery, The First Affiliated Hospital of Xi’an Jiaotong University, Xi’an 710061, China; docliqi@163.com (Q.L.); tangqingyu@stu.xjtu.edu.cn (Q.T.); 18375762570@163.com (D.X.); liu200405@stu.xjtu.edu.cn (H.L.); tangzhq18@163.com (Z.T.); zhangdong811021@126.com (D.Z.)

**Keywords:** gallbladder cancer, lymph node metastasis, gene mutations, DEGs, SMGs, *PLCL2*

## Abstract

**Background/Objectives**: Gallbladder cancer (GBC) is an aggressive biliary tract malignancy with poor prognosis. Lymph node metastasis is a major determinant of adverse outcome in patients with GBC. Gene mutations play an essential role in tumorigenesis; however, it remains uncertain whether genetic mutations play a substantial role in lymph node metastasis in GBC. **Methods**: In this study, transcriptome and whole-exome sequencing (WES) were used to analyze gene mutations and expression in GBC tissues, focusing on lymph node metastasis. Bioinformatics tools identified differentially expressed genes (DEGs) and significantly mutated genes (SMGs), followed by pathway enrichment and survival analyses. **Results**: In total, 669 DEGs were identified between metastatic and non-metastatic GBC tissues. Through protein–protein interaction (PPI) network analysis of these DEGs, *GPT* and *NR1I2* were identified as candidate genes associated with metabolic reprogramming in lymph node metastasis. Prognostic analysis revealed 22 DEGs associated with patient survival, and significant differences in overall survival, clinicopathological features (e.g., N-stage and positive lymph node count) were observed between cluster 1 and cluster 2. Mutation analysis identified 55 SMGs, primarily related to immune and inflammatory responses. By integrating DEGs and SMGs, *PLCL2* was identified as a candidate gene potentially associated with both lymph node metastasis and prognosis. GSEA enrichment analysis suggested that *PLCL2* was potentially linked to immunity, inflammation, and cellular processes, which may imply its possible involvement in GBC metastasis pending experimental validation. **Conclusions**: Based on integrative transcriptomic and exomic analyses, we identified *PLCL2* as a candidate gene potentially associated with lymph node metastasis in GBC. These hypothesis-generating findings provide a preliminary basis for future mechanistic validation and biomarker exploration.

## 1. Introduction

Gallbladder cancer (GBC) is the most common malignancy of the biliary system. Globally, the incidence of GBC is significantly higher in females than in males, and there is a marked regional variation [1,2]. Gallstones are the primary risk factor for GBC [3], accompanied by various other environmental and genetic predisposing factors [4,5]. GBC lacks specific early symptoms, with two-thirds of early cases being incidental findings and ~50% presenting with advanced-stage disease at diagnosis [6,7]. In recent years, significant progress has been made in multidisciplinary treatments such as immunotherapy, targeted therapy, chemotherapy, and local therapy for GBC [8]. Currently, radical resection remains the only potential curative approach for GBC, but lymph node metastasis significantly limits the success rate of surgery and the prognosis of patients. Exploring the mechanisms of lymph node metastasis in GBC is crucial for developing new therapies, improving patient prognosis, and enhancing treatment outcomes.

Genetic alterations play a central role in tumorigenesis. In GBC, recurrent mutations have been identified in genes such as *TP53* and *KRAS* [9,10,11,12,13]. The incidence of mismatch repair deficiency and microsatellite instability is relatively low in GBC, occurring in less than 10% of cases [14]. High tumor mutational burden is uncommon in GBC, with an incidence rate ranging from 1.2% to 5.8% [15]. While genomic technologies have advanced our understanding of the genetic landscape of GBC, how these mutations specifically promote lymph node metastasis remains a critical knowledge gap that needs to be addressed.

RNA sequencing (RNA-seq) directly captures gene expression profiles, whereas whole-exome sequencing (WES) enables the identification of potential mutations that may influence gene expression regulation, including variants located in regulatory regions or associated with transcription factor binding, thereby affecting transcriptional activity [16]. However, joint analyses of transcriptomic and WES data to characterize the mutational landscape underlying lymph node metastasis in GBC remain scarce. Distinct from previous single-omics studies, we first integrated RNA-seq and WES to identify key candidates (e.g., *PLCL2*) via intersecting DEGs and significantly mutated genes (SMGs). This work provides a multidimensional molecular atlas of GBC lymph node metastasis, offering novel insights for targeted intervention.

Overall, this study characterized the transcriptomic alterations and mutational landscape of GBC with lymph node metastasis, identified potential candidate genes involved in this process, and may provide a basis for future mechanistic studies and biomarker validation.

## 2. Materials and Methods

### 2.1. Ethical Approval

This study was approved by the Ethics Committee of the First Affiliated Hospital of Xi’an Jiaotong University (No. XJTU1AF2022LSK-089), Xi’an, China. Written informed consent was obtained from all included patients and their families before study enrollment.

### 2.2. Sources of Data

Between August 2020 and September 2022, a total of 37 patients with pathologically confirmed GBC treated at the First Affiliated Hospital of Xi’an Jiaotong University were retrospectively included in this study (Supplementary Table S1). All patients underwent radical-intent surgery, and lymph node status was determined by postoperative histopathological examination of regional lymph nodes. According to the AJCC 8th edition TNM staging system, patients were classified as N0 (no regional lymph node metastasis), N1 (1–3 metastatic regional lymph nodes), or N2 (≥4 metastatic regional lymph nodes). Based on this criterion, 17 patients were assigned to the non-metastasis group (N0) and 20 patients to the lymph node metastasis group (N1/N2). N1 and N2 cases were combined for downstream analyses because both represent pathologically confirmed regional lymph node metastasis, and the limited sample size did not support stable subgroup analyses.

Inclusion criteria were as follows: (1) pathologically confirmed GBC; (2) clearly documented postoperative lymph node status; (3) availability of sufficient tumor tissue for sequencing; and (4) no preoperative radiotherapy or chemotherapy. Exclusion criteria were: (1) poor nucleic acid quality; (2) insufficient tumor tissue for sequencing; and (3) incomplete clinicopathological data. All 37 cases had both RNA-seq and WES data available. Tumor tissues were used for sequencing analyses. DNA for WES was extracted from FFPE tumor tissues, whereas RNA for RNA-seq was extracted from tumor tissues using TRIzol reagent, with only samples meeting the RNA quality criterion (RIN > 7.0) included for library construction.

### 2.3. RNA Extraction, Sequencing, and Bioinformatics Analysis

Total RNA was extracted from tumor tissues using TRIzol reagent (Thermo Fisher, Wilmington, DE, USA) according to the manufacturer’s instructions. RNA quantity and purity were assessed using a NanoDrop ND-1000 spectrophotometer (Thermo Fisher, DE, USA), and RNA integrity was evaluated using an Agilent 2100 Bioanalyzer (Agilent Technologies, Santa Clara, CA, USA). Only samples with RNA integrity number (RIN) > 7.0 were included. Poly(A)+ RNA was isolated from 1 μg of total RNA using Dynabeads Oligo (dT) (Thermo Fisher, Wilmington, DE, USA), fragmented, reverse-transcribed into cDNA, ligated to adaptors, size-selected using AMPure XP beads, and amplified by PCR. Libraries with an average insert size of approximately 300 ± 50 bp were sequenced on the Illumina NovaSeq 6000 platform (LC-Bio Technology, Hangzhou, China) in paired-end 150 bp mode. Raw sequencing reads were processed using fastp (v0.23.4) for adapter trimming and quality filtering with default parameters. GRCh38 was used as the reference genome because its improved genome representation and annotation, compared with earlier genome builds, facilitate more accurate read alignment, gene expression quantification, and transcript identification [17]. Clean reads were aligned to the human reference genome GRCh38 using HISAT2 (v2.2.1) with the --dta option. Gene annotation was based on GENCODE v44, and transcript assembly and expression quantification were performed using StringTie (v2.2.1). Gene expression levels were normalized as fragments per kilobase of transcript per million mapped reads (FPKM).

### 2.4. DNA Extraction, Sequencing, and Bioinformatics Analysis

Genomic DNA was extracted from tumor tissues using the QIAamp DNA FFPE Tissue Kit (QIAGEN, Hilden, Germany) according to the manufacturer’s protocol. DNA libraries were prepared using the Covaris M220 Focused Ultrasonic System, and exonic regions were captured using a SureSelect Human All Exon V6 kit (Agilent Technologies, Santa Clara, CA, USA). Sequencing was performed on the Illumina NovaSeq 6000 platform (LC-Bio Technology, Hangzhou, China) in paired-end 150 bp mode. Raw reads were processed using Trimmomatic (v0.39) and FastQC (v0.11.9) for adapter trimming and quality filtering. Clean reads were aligned to the human reference genome hg19 using BWA (http://maq.sourceforge.net, accessed on 1 May 2026) with default parameters [18,19]. The hg19 reference genome was selected because it has been widely adopted in whole-exome sequencing studies and is broadly compatible with established variant databases and bioinformatics tools, thereby facilitating variant annotation, cross-study comparison, and result validation [20]. PCR duplicates were removed using Picard (http://broadinstitute.github.io/picard/, accessed on 1 May 2026). Single-nucleotide variants (SNVs) and small insertions and deletions were called using Mutect (https://github.com/broadinstitute/mutect, accessed on 1 May 2026) and Strelka2 (https://github.com/Illumina/strelka, accessed on 1 May 2026) under a tumor-only workflow, because matched normal tissue or peripheral blood samples were not available. Functional annotation was performed using VEP (Ensembl release 110) [21,22,23]. Copy number variation (CNV) analysis was conducted using Control-FREEC with GC-content normalization under a tumor-only setting [24].

### 2.5. Identification of DEGs and SMGs

To identify genes exhibiting differential expression between the metastatic and non-metastatic groups within the transcriptome dataset, the R package “DESeq2” was employed to detect DEGs through transcriptome sequencing [25]. DEGs were identified using criteria wherein raw *p* < 0.05 and |log2Fold Change| > 1. The density heatmaps of these DEGs were displayed using ComplexHeatmap [26]. To investigate the genes that were significantly mutated in each sample, we used the oncodrive function in the R package “maftools” (version 2.14.0, https://github.com/PoisonAlien/maftools/issues, accessed on 1 May 2026), and the mutated genes that affected the functional domain could be used as candidate cancer driver genes. Moreover, some studies also showed that the high-frequency mutations and known driver genes had a clear functional mutation preference. Here, the oncodrive function was used and all samples in the GBC metastasis group were screened according to the threshold FDR < 0.05 and marked as significantly mutated genes (SMGs).

### 2.6. GO and KEGG Pathway Enrichment Analysis

To explore the gene functions and biological processes in which candidate genes were involved, the R package “clusterProfiler” (version 4.8.3) was employed to perform Gene Ontology (GO) and Kyoto Encyclopedia of Genes and Genomes (KEGG) enrichment analyses on the candidate genes. GO terms were categorized into Biological Process (BP), Cellular Component (CC), and Molecular Function (MF). Pathway enrichment analysis aimed to identify relevant biological functions and signaling pathways with an adjusted *p* < 0.05.

### 2.7. Building Protein–Protein Interaction (PPI) Networks and Identifying Hub Genes

To explore the interactions of candidate genes at the protein level, the candidate genes were entered into the STRING database (https://string-db.org/, accessed on 1 May 2026) to construct a PPI network, and this network was visualized using Cytoscape (interaction score = 0.4). The results were realized by Cytoscape software (version 3.8.2) [27]. Subsequently, seven algorithms (Maximum Neighborhood Component (MNC), Degree, Edge Percolated Component (EPC), betweenness, Closeness, Radiality and BottleNeck) were employed to identify the hub genes in the PPI networks. Hub genes were determined by selecting those genes that appeared in the top 10 rankings across all seven algorithms.

### 2.8. Screening for Prognostic Genes

Overall survival (OS) was defined as the interval from the date of surgery to the date of death from any cause or last follow-up. Survival analyses were performed within the lymph node metastasis subgroup (*n* = 20). To screen prognosis-associated genes within the lymph node metastasis cohort, univariable Cox proportional hazards regression was performed for each DEG using the R package “Survival”. Genes with hazard ratio (HR) ≠ 1 and raw *p* < 0.05 were retained as candidate prognosis-related genes for downstream consensus clustering and exploratory survival analyses.

### 2.9. Consensus Cluster Analysis

Employing prognostic-related genes, consensus cluster analysis was executed via the R package “ConsensusClusterPlus” to stratify lymph node metastasis patients. Consensus clustering was performed on 20 patients with lymph node metastasis using 22 prognostic-related genes. Parameters were Euclidean distance, hierarchical clustering (complete linkage), and 1000 iterations with 80% resampling. Optimal cluster number was determined by CDF curves and delta area. Following that, principal component analysis (PCA) was conducted with the R software package “FactoMineR” (version 2.9) to validate the classification. Kaplan–Meier (KM) survival analysis was performed for different clusters utilizing the R packages “Survival” and “Survminer” to assess differences in prognosis among various clusters. Subsequently, the Wilcoxon test was employed to explore correlations between clusters and clinicopathological characteristics (age, stage, N_Stage, T_Stage, M_Stage, gallstone, gallbladder polyps, gender, liver invasion, perineural invasion, pathological differentiation degree, positive lymph node count), analyzing significant differences in clinicopathological characteristics between different clusters.

### 2.10. Comprehensive Analysis of Somatic Mutations in GBC

To investigate the distribution of somatic mutations in patients with GBC lymph node metastasis using WES data, the R package “Maftools” was used to generate and analyze the histograms of somatic mutations in two clusters [28].

### 2.11. Analysis of the Variation Landscape of DEGs

To identify the mutant landscape of DEGs between the 2 cluster groups, the mutations of DEGs in metastatic and non-metastatic samples from patients from each cluster were assessed using the R package “Maftools” [28]. The mutations of DEGs in WES data from the 2 clusters were scrutinized, and the mutational landscape of the top 20 DEGs with mutations was shown. We employed the R package “sigminer” to analyze the mutation features of mutated genes across diverse populations based on exon data [29]. We generated two clusters of somatic SNV mutation matrices and extracted their characteristics. The six variants (C > A, C > G, C > T, T > A, T > C and T > G) of a mutant base were analyzed using the single base replacement (SBS) method to determine the mutation types. Subsequently, we extracted and visualized clustering for catastrophic patterns based on COSMIC annotations (v. 3.2, https://cancer.sanger.ac.uk/signatures/sbs/, accessed on 1 May 2026).

### 2.12. Screening and Enrichment Analysis of SMGs

We screened to capture the significantly mutated gene in each sample of the WES data according to the oncodrive function in maftools. Predictions were made based on the virulence of functional mutations, based on the DEGs of the GBC lymph node metastasis group, according to a threshold FDR < 0.05 to screen SMGs. Additionally, to delve deeper into the potential role and function of SMGs and their impact on pathways, the R package “clusterProfiler” was employed for GO function analysis and KEGG enrichment analysis. The SMGs were inputted into the STRING database (https://string-db.org/, accessed on 1 May 2026) to construct the PPI network (confidence level = 0.4). The outcomes were achieved through Cytoscape software.

### 2.13. Key Gene Identification and Gene Set Enrichment Analysis (GSEA)

To further investigate the relevant underlying biological mechanisms of key genes, the R package “ggvenn” (version 0.1.10) was used. The intersection of DEGs and SMGs was used to obtain key genes for subsequent analysis. GSEA pathway enrichment analysis was performed using the R package “clusterProfiler” (version 4.83). The threshold FDR < 0.05 was considered significantly enriched.

### 2.14. Analysis of the Key Genes’ Survival and Mutation Site

In order to explore the potential prognostic significance of key genes, this study divided all samples from the lymph node metastasis group into a high-expression group (3 samples) and a low-expression group (17 samples) according to the optimal cut-off value (0.4930528). Subsequently, the R package “Survival” was employed to generate survival curves for patients in both groups, aiming to examine the association between key gene expression levels and patient survival. Moreover, in order to further analyze the mutational site of key genes on peptide sequences, the Lollipops tool was used to map the mutational site of key genes.

### 2.15. Analysis of Immune Cell Infiltration

In order to study the differences in immune cell infiltration between different samples (cluster 1 and cluster 2), we conducted an analysis through ssGSEA (version 1.46.0) [30]. The differences in immune-infiltrating cells between the samples of cluster 1 and cluster 2 were compared by Wilcoxon test (*p* < 0.05), and box plots were drawn using “ggplot2” (version 3.5.1) for display [31]. To further explore the relationship between the differential immune cells, the “cor” function in the R package (version 3.5.1) was used to evaluate the correlations among the differential immune cells through Spearman analysis [32]. The immune checkpoints were sourced from references, and the differential expression of common immune checkpoints in cluster 1 vs. cluster 2 was further analyzed, and a correlation analysis between the differential immune checkpoints and prognostic genes was carried out [33,34].

### 2.16. Statistical Analyses

All analyses were performed using R software (version 4.2.2). Differences between groups were analyzed by Wilcoxon test, and *p* < 0.05 was considered statistically significant.

## 3. Results

### 3.1. Screening and Functional Enrichment Analysis of DEGs and Hub Gene Acquisition

**Figure 1:**
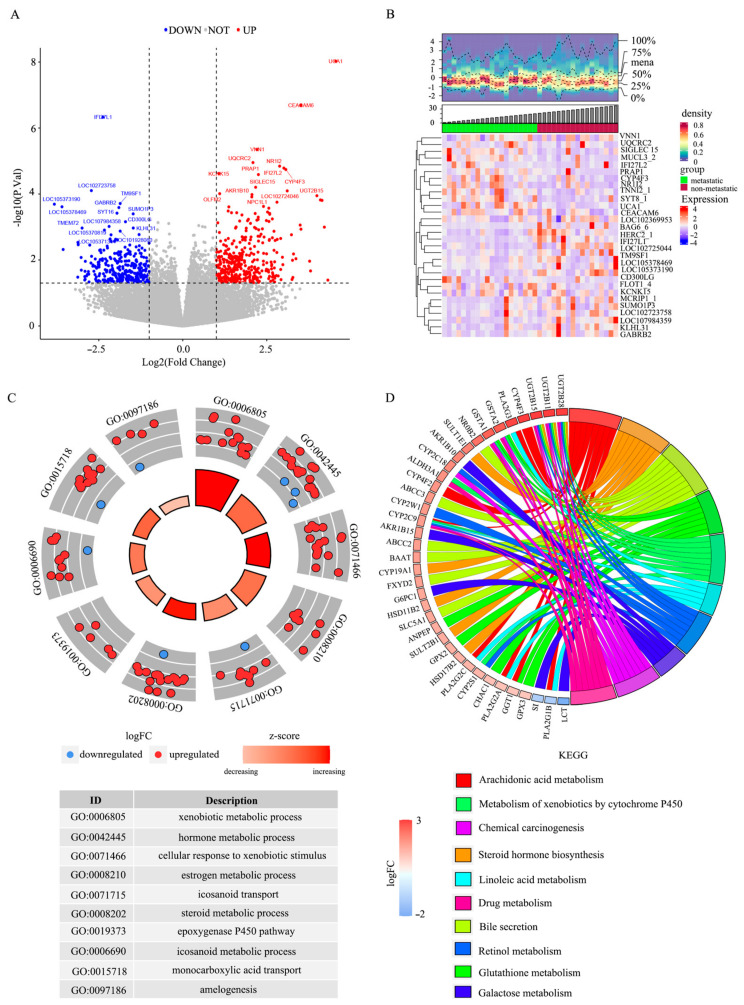
Screening and functional enrichment analysis of DEGs. (**A**) Volcano plot of DEGs. (**B**) Density heatmap of DEGs. (**C**) Top 10 GO enrichment analysis of DEGs. (**D**) Top 10 KEGG enrichment analysis of DEGs. Different colors were used to distinguish different pathways, and the genes pointed to by the chords were the genes enriched in those pathways.

Based on the GBC transcriptome dataset, 669 DEGs were identified in both the lymph node metastatic and the non-metastasis GBC. Of these, 394 DEGs were up and 275 DEGs were down in the GBC lymph node metastasis group (Figure 1A). The top 30 upregulated and top 30 downregulated DEGs are shown in the heatmap (Figure 1B). GO enrichment analysis indicated that these DEGs were mainly involved in xenobiotic metabolism and hormone-related transcriptional processes (Figure 1C). KEGG analysis showed enrichment in pathways related to steroid hormone biosynthesis and other metabolic processes (Figure 1D). This study constructed PPI networks consisting of 236 nodes and 541 edges. Candidate hub genes *GPT* and *NR1I2* were identified through PPI network topology, ranking within the top 10 across all seven algorithms (Figure S1), suggesting their potential involvement in metastatic biology pending functional validation.

### 3.2. Identification and Classification of Prognostic Genes

**Figure 2:**
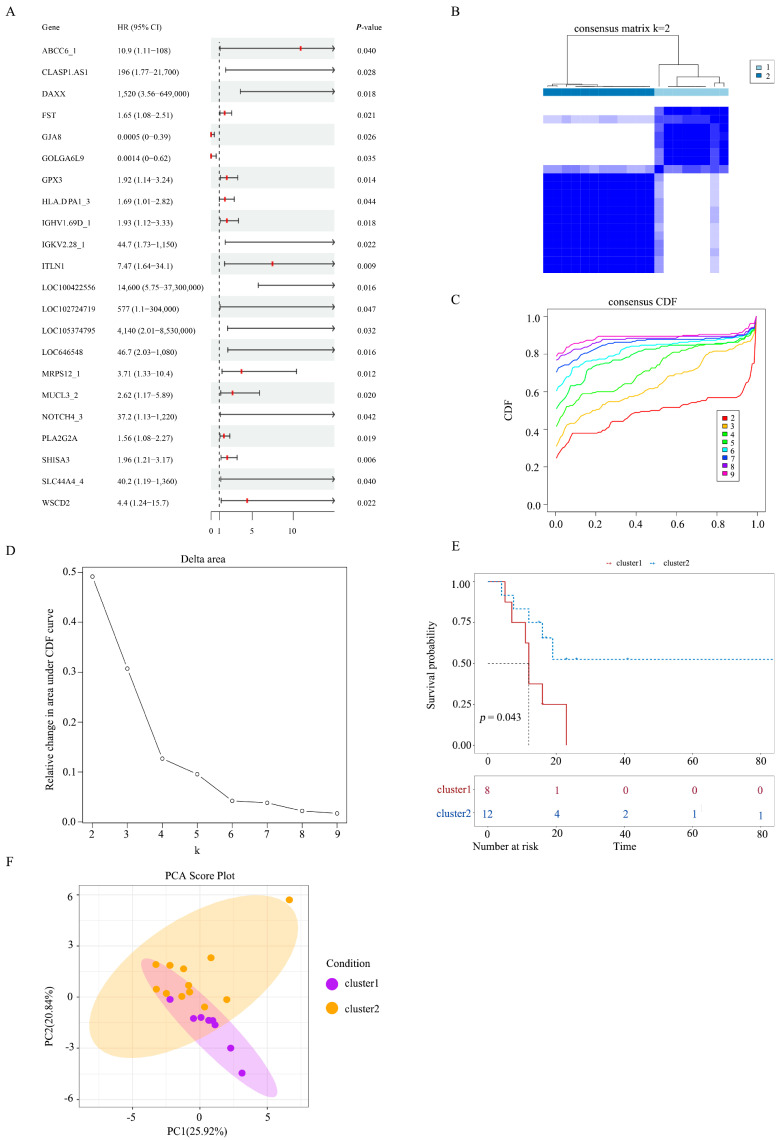
Identification and classification of prognostic genes. (**A**) Forest plot for prognosis-related DEGs. Forest plot shows the result of multivariate Cox regression between DEGs and prognosis. (**B**) Consensus matrix diagram for samples of lymph node metastasis cohort. (**C**) CDF curve for samples of lymph node metastasis cohort. (**D**) Delta area plot for determining optimal number of clusters. (**E**) Kaplan–Meier survival curve comparison chart between cluster 1 and cluster 2. (**F**) PCA plot for samples of lymph node metastasis cohort.

Identification and classification were performed through multivariate Cox regression analysis of 669 DEGs, 22 of which were prognostic-related genes (HR > 1, *p* < 0.05) (Figure 2A). Additionally, systematic clustering analysis was performed on prognosis-related genes, yielding two clusters (Figure 2B–D). The KM survival analysis found significant differences between cluster 1 and cluster 2 (Figure 2E), with cluster 1 comprising 8 samples and cluster 2 consisting of 12 samples (Figure 2F). Beyond N-stage and positive lymph node count, differences in clinicopathological characteristics were observed between the two clusters (Figure S2): cluster 1 was characterized by T4 stage, poor differentiation, liver invasion, and perineural invasion, whereas cluster 2 predominantly exhibited T3 stage, moderate differentiation, and absence of invasion. These disparities align with molecular findings: the aggressive phenotype of cluster 1 corresponds to its enriched hub genes for metabolic reprogramming (*GPT*, *NR1I2*) and TP53 mutations; the lower aggressiveness of cluster 2 is consistent with its immune/inflammation-related SMGs (e.g., MHC pathway genes) and correlates with the survival trend observed in the *PLCL2* high-expression group.

### 3.3. Comprehensive Analysis of GBC Mutation

**Figure 3:**
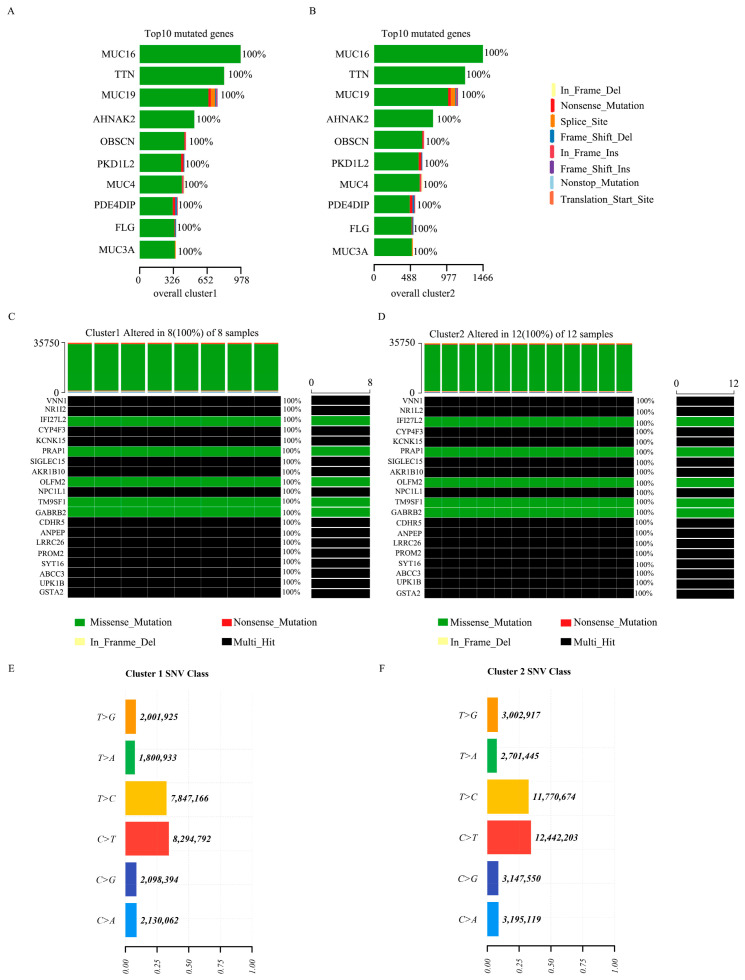
Comprehensive analysis of GBC mutation. (**A**) Histogram of the top 10 mutated genes in cluster 1. (**B**) Histogram of the top 10 mutated genes in cluster 2. (**C**) Distribution map of mutation types for the top 20 DEGs in cluster 1. Black indicates multiple mutations, while green indicates only missense mutations. (**D**) Distribution map of mutation types for the top 20 DEGs in cluster 2. (**E**) SNV class in cluster 1. (**F**) SNV class in cluster 2.

The histogram of the somatic mutation distribution of GBC patients in cluster 1 and cluster 2 showed that in both clusters, MUC16 produces the largest number of mutation samples and the most missense mutation types. The second is TTN, where the top ten mutated genes in both clusters were the same and mutated in all samples (Figure 3A,B). Mutation analysis in the 669 DEGs showed that 249 genes with mutations were detected in the exome data. Mutations were observed in all patients in cluster 1 and cluster 2. The mutations of the top 20 DEG were visualized (Figure 3C,D). In the analysis of SNV, it was found that mutations in both clusters predominantly involved C > T and T > C (Figure 3E,F).

### 3.4. Identify SMGs with Significant Mutations

**Figure 4:**
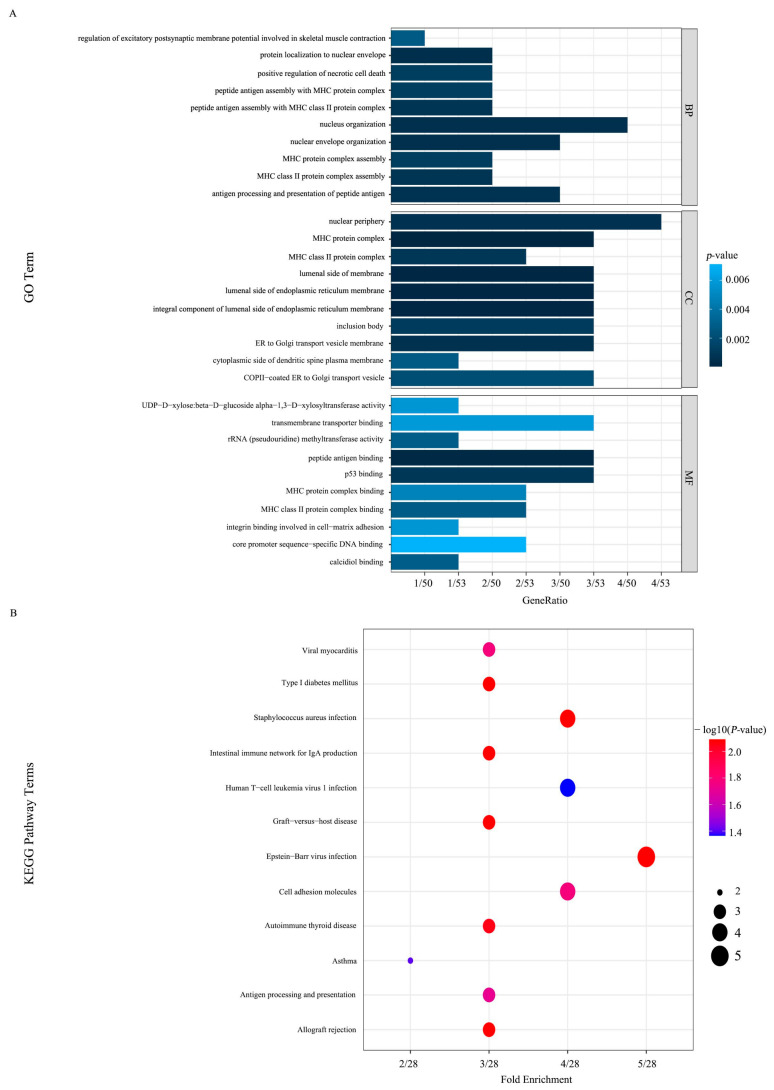
Functional enrichment analysis of SMGs. (**A**) GO enrichment analysis of SMGs. (**B**) KEGG enrichment analysis of SMGs.

A total of 55 SMGs were identified in the GBC lymph node metastasis group, and multiple mutations occurred in all samples (Figures S3 and S4). The GO enrichment analysis of SMGs showed 263 BP, 52 CC and 75 MF entries. These SMGs were primarily associated with the function of MHC proteins, which are vital components of the immune system and are closely related to inflammatory processes in tumors (Figure 4A). SMGs were found to be enriched in 22 KEGG pathways. The outcomes revealed a predominant annotation of pathways associated with immune inflammatory responses, consistent with the findings of GO enrichment analysis (Figure 4B). A PPI network of the SMGs was constructed, comprising 21 nodes and 20 edges, with TP53 identified as the key gene (Figure S5).

### 3.5. Identification and Enrichment of DEGs with Significant Mutations

**Figure 5:**
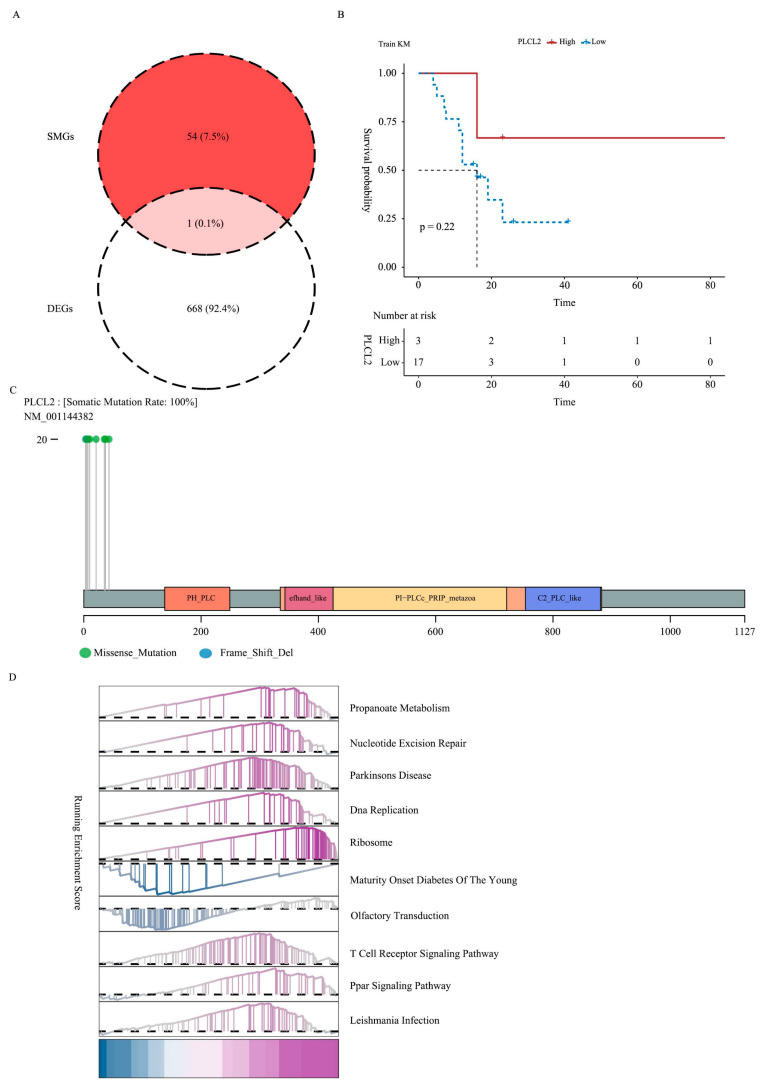
Identification and enrichment of DEGs with significant mutations. (**A**) Venn diagram illustrating the intersection of SMGs and DEGs. (**B**) The mutation site map of *PLCL2*. (**C**) Kaplan–Meier survival curve comparison chart between high-*PLCL2* group and low-*PLCL2* group. (**D**) Immune-related signaling pathways enriched by GSEA analysis of *PLCL2* gene.

The intersection of DEGs and SMGs resulted in a significantly mutated differential expression of *PLCL2* (Figure 5A). The mutation site map of *PLCL2* was drawn to show the mutation site (Figure 5B). Utilizing *PLCL2* expression, the GBC lymph node metastasis group was stratified into two categories: a group with high expression (3 samples) and a group with low expression (17 samples). The Kaplan–Meier survival curve indicated a trend toward poorer survival in the low-expression group (Figure 5C). GSEA of *PLCL2* expression suggested enrichment of pathways potentially related to immunity and inflammation (e.g., TCR signaling, PPAR signaling), alongside other processes (DNA replication, ribosomal pathways, olfactory transduction) (Figure 5D), though causal links require experimental confirmation.

### 3.6. Differences in Immune Cell Infiltration and Immune Checkpoints Among Different Clusters

**Figure 6:**
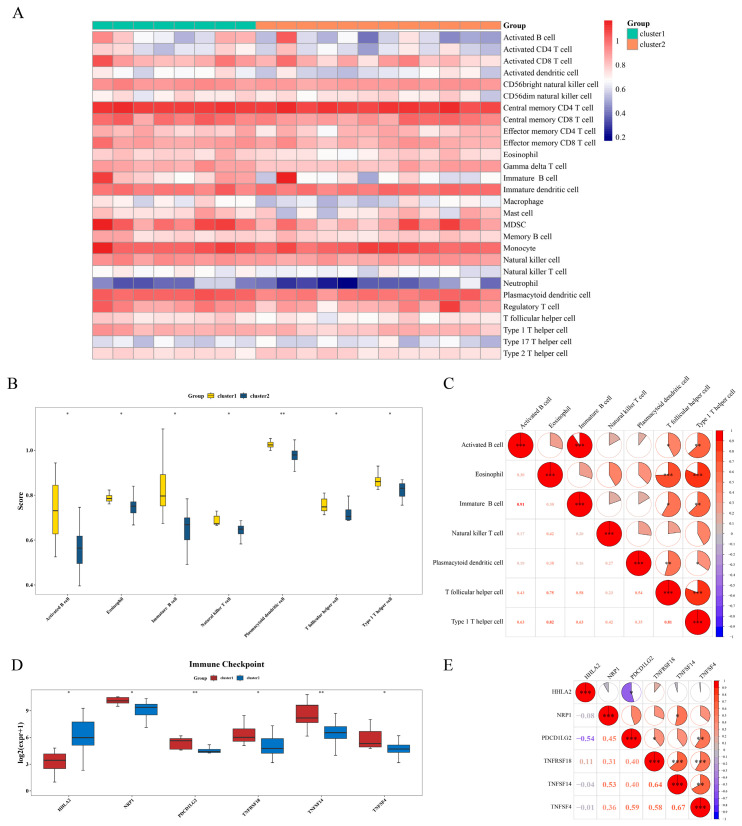
Analysis of immune cell infiltration and immune checkpoints. (**A**) The proportions of immune-infiltrating cells in different samples of the training set. (**B**) The differences of immune-infiltrating cells in different samples. (**C**) Correlation analysis between differential immune cells and correlation analysis between biomarkers and differential immune cells. The scores represent correlations. (**D**) Differential expression analysis of common immune checkpoints in cluster 1 vs. cluster 2 groups. (**E**) Correlation analysis between differential immune checkpoints and prognostic genes. The scores represent correlations; the size of the circle area indicates significance, with red representing positive correlations and blue representing negative correlations. Among them, *, *p* < 0.05; **, *p* < 0.01; ***, *p* < 0.001.

Firstly, an immune infiltration analysis was performed for 28 types of immune cells (Figure 6A). Then, the differences in immune infiltrating cells between the samples of cluster 1 and cluster 2 were compared (*p* < 0.05). Subsequently, the immune scores of these seven immune cells (activated B cell, eosinophil, immature B cell, natural killer T cell, plasmacytoid dendritic cell, T follicular helper cell, type 1 T helper cell) were obtained (Figure 6B). Furthermore, we also evaluated the correlations among the differential immune cells (cor > 0.3, *p* < 0.05). Among the immune cells, the correlation coefficient between activated B cell and immature B cell was the highest, reaching 0.91 with *p* < 0.05 (Figure 6C). There were a total of six immune checkpoints with significant differences between the cluster 1 and cluster 2 groups (Figure 6D). Among the immune checkpoints, TNFSF14 and TNFSF4 had the highest correlation, with a correlation coefficient of 0.67 and *p* < 0.05 (Figure 6E).

## 4. Discussion

Lymph node metastasis in GBC indicates that cancer cells have breached the local region and spread to nearby areas, increasing the treatment complexity and reflecting advanced disease progression with poor prognosis. While prior studies have reported individual gene mutations in GBC, few have integrated transcriptomic and exomic data to systematically explore the mutational landscape underlying lymph node metastasis [9,10,11,12,13]. Our study is the first to identify *PLCL2* as a candidate associated with both lymph node metastasis and prognosis via multi-omics intersection. Against this backdrop, we analyzed potential mechanisms by which gene mutations promote GBC lymph node metastasis using integrated transcriptome and WES approaches.

In this study, we identified 669 DEGs from transcriptome data of lymph node metastatic and non-metastatic GBC. GO and KEGG analyses showed that the DEGs were primarily enriched in processes such as steroid hormone metabolism, eicosanoid metabolism, linoleic acid metabolism, and arachidonic acid metabolism. This study found that the key metabolic product of cholesterol metabolism, 27-hydroxycholesterol, leads to excessive lipid accumulation by increasing lipid uptake or synthesis in cells, upregulating the expression of glutathione peroxidase 4 (GPX4), which makes breast cancer cells resistant to ferroptosis [35]. Research has shown that the metabolic profile of breast cancer patients significantly differs from that of healthy controls, particularly in lipid metabolism and steroid hormone metabolism. Targeted analysis of steroid hormones and their metabolites can reveal key metabolic changes during tumor development and aid in disease classification, prognosis assessment, and treatment decision-making [36]. Another study discovered that FADS1 promotes the synthesis of arachidonic acid, alters gut microbiota, and increases the abundance of Gram-negative bacteria. These bacteria further activate the TLR4/MYD88 signaling pathway, promoting the metabolism of arachidonic acid to prostaglandin E2 (PGE2), thereby driving the occurrence and development of colorectal cancer [37]. In liver cancer research, ENO1 was found to promote the translation of YAP1 by binding to YAP1 mRNA. YAP1 activates arachidonic acid metabolism by regulating the expression of PLCB1 and HPGD, leading to the accumulation of PGE2, which promotes tumor growth and progression [38].

The DEG-derived PPI network highlighted *GPT* and *NR1I2* as hub genes, suggesting that metabolic reprogramming may be involved in the biology of lymph node metastasis in GBC. *GPT* encodes glutamic-pyruvic transaminase 1, which reversibly catalyzes the conversion of alanine and α-ketoglutarate into pyruvate and glutamate, thereby playing a key role in amino acid metabolism and glucose intermediate metabolism [39]. During tumor metastasis and chemoresistance, cancer cells must rewire their bioenergetic metabolism. *GPT* may contribute to this process by modulating α-ketoglutarate (α-KG) levels, thereby influencing HIF signaling and DNA demethylation to drive EMT and metastatic colonization [40,41]. The *NR1I2* gene encodes the pregnane X nuclear receptor, which regulates the expression of drug-metabolizing enzymes and transport proteins, playing a role in drug detoxification as well as cholesterol, bile acid, and lipid metabolism. In digestive malignancies such as liver cancer, elevated *NR1I2* orchestrates a multifaceted pro-tumorigenic program. Beyond driving chemoresistance through the induction of metabolic enzymes like CYP3A4, *NR1I2* remodels the tumor microenvironment by regulating lipid and bile acid metabolism (e.g., CYP7B1, CYP8B1). Crucially, its physical interaction with transcription factors like ETS-1 directly fuels the EMT process and metastatic invasion [42,43,44]. Given that our GO/KEGG analyses converged on steroid- and lipid-related metabolic pathways, the emergence of *GPT* and *NR1I2* as hub genes is biologically plausible. However, these genes were identified through network topology without direct mechanistic validation. Therefore, we consider them candidate hub genes potentially linked to metastatic biology.

Gene mutations drive tumorigenesis and progression, affecting cancer cell proliferation and drug resistance. Analyzing gene mutations helps identify tumor drivers and personalize treatment strategies. In this study, mutation analysis of patients in the lymph node metastasis group revealed that MUC16 had the most mutated samples and the most missense mutations, followed by TTN. The MUC16 gene encodes a large mucin, commonly known as CA125, a cell-surface glycoprotein widely expressed in epithelial cells. Under normal physiological conditions, MUC16 helps protect epithelial cells, but its expression is significantly increased in cancers. MUC16 gene mutations have been found in various cancers, especially ovarian cancer. These mutations typically affect the structure and function of the MUC16 protein, leading to enhanced proliferation, adhesion, migration, and immune evasion of tumor cells. One study suggested that the MUC16 gene promotes liver metastasis in pancreatic ductal adenocarcinoma (PDAC) by regulating NRP2 expression through the activation of the JAK2/STAT1 signaling pathway, playing a critical role in the interaction between cancer cells, vascular endothelial cells, and platelets during PDAC metastasis [45]. MUC16 also significantly promotes the progression and metastasis of PDAC by regulating cytoskeletal reorganization proteins and the tumor microenvironment [46]. In thyroid cancer research, TTN gene mutations were found to be significantly associated with poor prognosis in thyroid cancer (THCA) patients, suggesting that TTN gene mutations may serve as an independent risk factor for THCA and an important prognostic marker [47]. Other studies have shown that TTN gene mutations were significantly associated with tumor response in breast cancer patients receiving neoadjuvant chemotherapy, with TTN mutations linked to higher mutational burden and higher treatment response rates, suggesting that TTN mutations may serve as an important biomarker for predicting neoadjuvant chemotherapy response in breast cancer patients [48]. Due to their large size and high mutational burden, MUC16 and TTN are often considered potential “passenger mutations” rather than definitive drivers of GBC metastasis. However, prior studies suggest functional roles for these genes [40,42,43]. Therefore, further in vitro functional assays are required to clarify whether these mutations act as drivers or passengers in GBC metastasis.

To further investigate the mutational characteristics of the exome data, SNV analysis was performed, and it revealed that C > T and T > C substitutions were the dominant mutation types in both clusters. SNVs, as a common form of genetic alteration, may influence the function of metastasis-related genes and thus contribute to the molecular basis of lymph node metastasis [49]. For instance, SNVs in key cell-cycle regulators, including members of the CDK family, may alter protein activity and accelerate cell proliferation [50], thereby increasing the pool of tumor cells with metastatic potential [51]. Likewise, SNVs may impair the tumor-suppressive function, allowing genetically abnormal cells to evade apoptosis and continue progressing toward a more aggressive phenotype [49]. Therefore, SNV analysis may help identify functionally altered genes associated with metastatic progression and provide important insights into the molecular mechanisms of lymph node metastasis in GBC [52].

In the lymph node metastasis group in GBC samples, we identified 55 significantly mutated SMGs and conducted GO and KEGG enrichment analyses, revealing the potential key roles of these SMGs in biological processes such as the immune system, cell adhesion, antigen processing, and presentation. Therefore, further research on the specific roles of these genes in GBC may provide important theoretical support for developing new immunotherapeutic strategies targeting GBC. By intersecting DEGs and SMGs, we identified *PLCL2* as a candidate gene associated with lymph node metastasis in GBC. In our cohort, *PLCL2* was both differentially expressed and significantly mutated, and GSEA suggested enrichment of immune- and inflammation-related pathways. Furthermore, studies have demonstrated that *PLCL2* exhibits functional hypermethylation in colorectal cancer, suggesting its potential role as a tumor suppressor gene in certain cancers [53]. Mechanistically, *PLCL2* links lipid metabolism to immune pathways via its C2 domain. By binding to membrane lipids, it acts as a scaffold to disrupt phospholipid metabolism and calcium homeostasis. This disruption activates the NF-κB and HIF-1α pathways, driving the release of pro-inflammatory cytokines (e.g., IL-6) and recruiting suppressive immune cells (e.g., TAMs). Thus, *PLCL2* likely fosters an immunosuppressive niche in GBC metastasis through lipid-driven signaling. These observations support a possible association between *PLCL2* and the metastatic tumor microenvironment in GBC. However, the biological directionality of this association remains unresolved. On the one hand, *PLCL2* was upregulated in the lymph node metastasis group. On the other hand, survival analysis within the metastatic cohort suggested a nonsignificant trend toward longer survival in the high-expression group. Given that the *PLCL2* high-expression subgroup included only three patients, this finding should be interpreted with substantial caution. In addition, the functional role of *PLCL2* in GBC cannot be inferred from retrospective bioinformatic association alone. Therefore, rather than concluding that *PLCL2* is a tumor suppressor gene or an immediately actionable therapeutic target, we consider it a potential biomarker that warrants validation in larger cohorts and GBC-specific mechanistic experiments. Future studies should validate *PLCL2* expression at the RNA and protein levels (e.g., qPCR, immunohistochemistry, or Western blot) and clarify its biological role in GBC using gain- and loss-of-function assays, invasion experiments, and in vivo metastasis models.

The increase in activated B cells reflects enhanced antigen presentation capacity in cluster 2, aligning with SMGs enriched in MHC and antigen processing pathways. Elevated eosinophils are typically associated with Th2-type inflammatory responses, potentially remodeling the tumor microenvironment via cytokine release. These immune profiles correlated with *PLCL2* expression patterns, suggesting *PLCL2* may indirectly influence immune infiltration by regulating inflammatory pathways.

This study has several important limitations. First, the overall cohort size was small (*n* = 37), and only 20 patients were included in the lymph node metastasis subgroup used for clustering, mutational characterization, and *PLCL2*-related survival analysis. In particular, the *PLCL2* high-expression group included only three patients, which substantially limited statistical power and made the survival comparison unstable. Therefore, the observed survival trend should be interpreted as exploratory rather than definitive. Second, the prognostic gene screening and subgroup analyses were performed in a relatively small retrospective cohort and require validation in larger independent datasets. Third, matched normal tissues or peripheral blood controls were not available for WES, which may have increased the risk of residual germline contamination despite downstream filtering. Fourth, no experimental validation was performed to confirm the expression pattern or biological function of *PLCL2* in GBC. Future studies should include larger multicenter cohorts, external validation, and GBC-specific functional experiments to verify the robustness and translational relevance of these findings.

In conclusion, integrated transcriptomic and exomic analyses identified *PLCL2* as a candidate metastasis-associated gene in GBC. Because these findings are based on a small retrospective cohort without functional validation, they should be considered exploratory and require confirmation in larger independent cohorts and GBC-specific experimental studies before any biomarker or therapeutic implications can be drawn.

## Data Availability

The data presented in this study are available on request from the corresponding authors due to privacy.

## Supplementary Materials

The following supporting information can be downloaded at: https://www.mdpi.com/article/10.3390/biomedicines14051076/s1, Figure S1: PPI networks of DEGs; Figure S2: Differences in clinicopathological features between cluster 1 and cluster 2; Figure S3: Distribution map of the 55 SMGs in cluster 1, with multiple mutations occurring in all samples; Figure S4: Distribution map of the 55 SMGs in cluster 2, with multiple mutations occurring in all samples; Figure S5: PPI networks of SMGs. Table S1: Baseline clinicopathological characteristics of the study cohort.

## Abbreviations

The following abbreviations are used in this manuscript:

BP	biological process
CC	cellular component
cDNA	complementary DNA
CNV	copy number variation
CRC	colorectal cancer
CSCs	cancer stem cells
DEGs	differentially expressed genes
DFS	disease-free survival
FDR	false discovery rate
FFPE	formalin-fixed paraffin-embedded
FPKM	fragments per kilobase of transcript per million mapped reads
GBC	gallbladder cancer
GC	guanine-cytosine
GO	Gene Ontology
GSEA	gene set enrichment analysis
HR	hazard ratio
KEGG	Kyoto Encyclopedia of Genes and Genomes
KM	Kaplan–Meier
MF	molecular function
MHC	major histocompatibility complex
OS	overall survival
PCA	principal component analysis
PCR	polymerase chain reaction
PDAC	pancreatic ductal adenocarcinoma
PGE2	prostaglandin E2
PI-PLC	phosphoinositide-specific phospholipase C
PPI	protein–protein interaction
RIN	RNA integrity number
RNA-seq	RNA sequencing
SBS	single base substitution
SMGs	significantly mutated genes
SNV	single-nucleotide variant
ssGSEA	single-sample gene set enrichment analysis
WES	whole-exome sequencing
