# Supplementary material for: Revealing the Potential Associations of Mutation-Related Genes with Lymph Node Metastasis in Gallbladder Cancer Through Transcriptome and Exome Sequencing
Source: Biomedicines. 2026 May 10;14(5):1076. doi: 10.3390/biomedicines14051076 (PMC13204380; doi:10.3390/biomedicines14051076)
Supplement: Supplementary file 1 [file biomedicines-14-01076-s001.zip › supplementary figures.pdf]

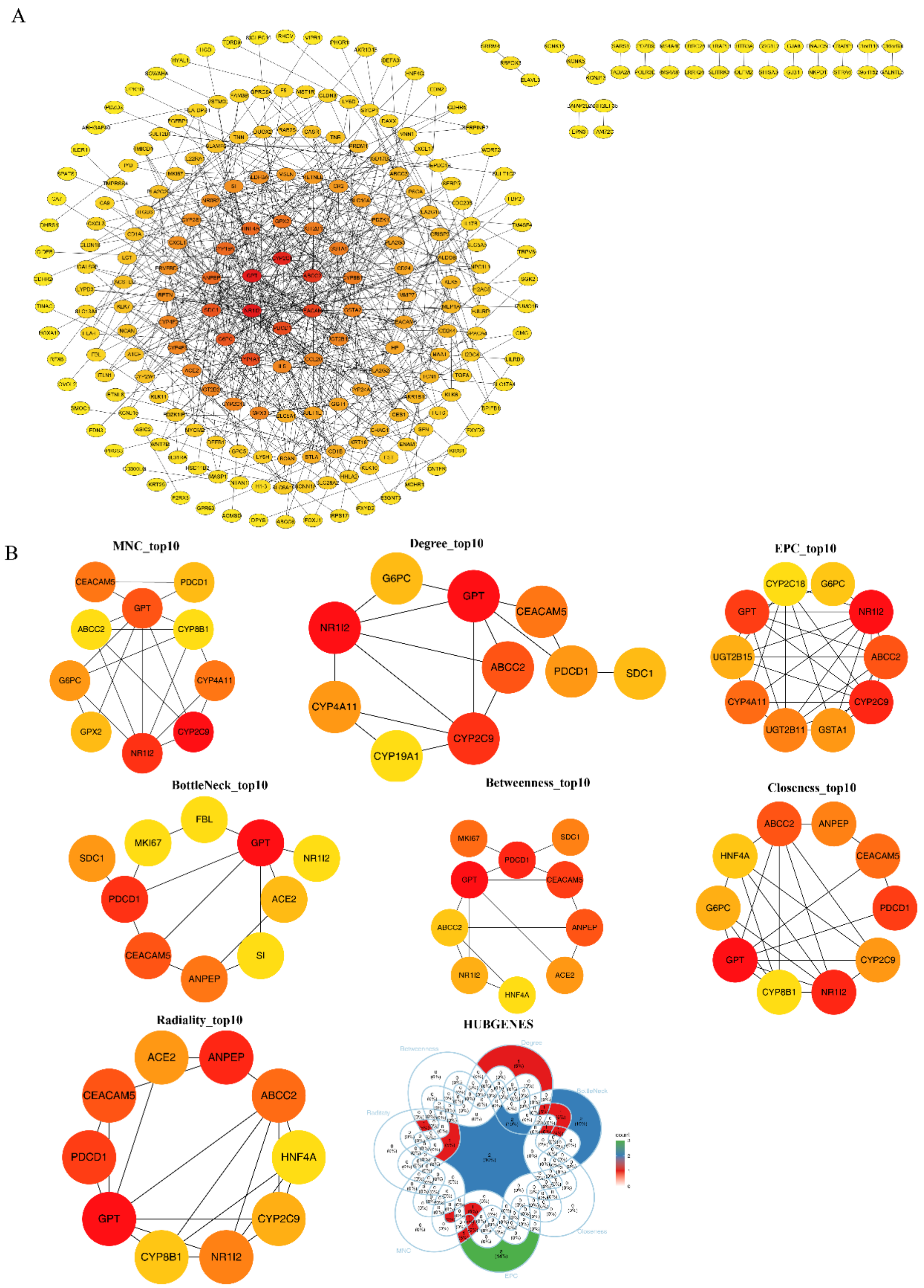

Figure S1. PPI networks of DEGs.

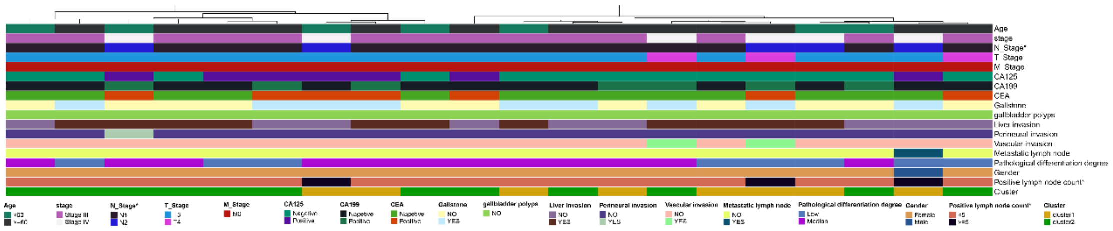

Figure S2. Differences in clinicopathological features between cluster 1 and cluster 2.

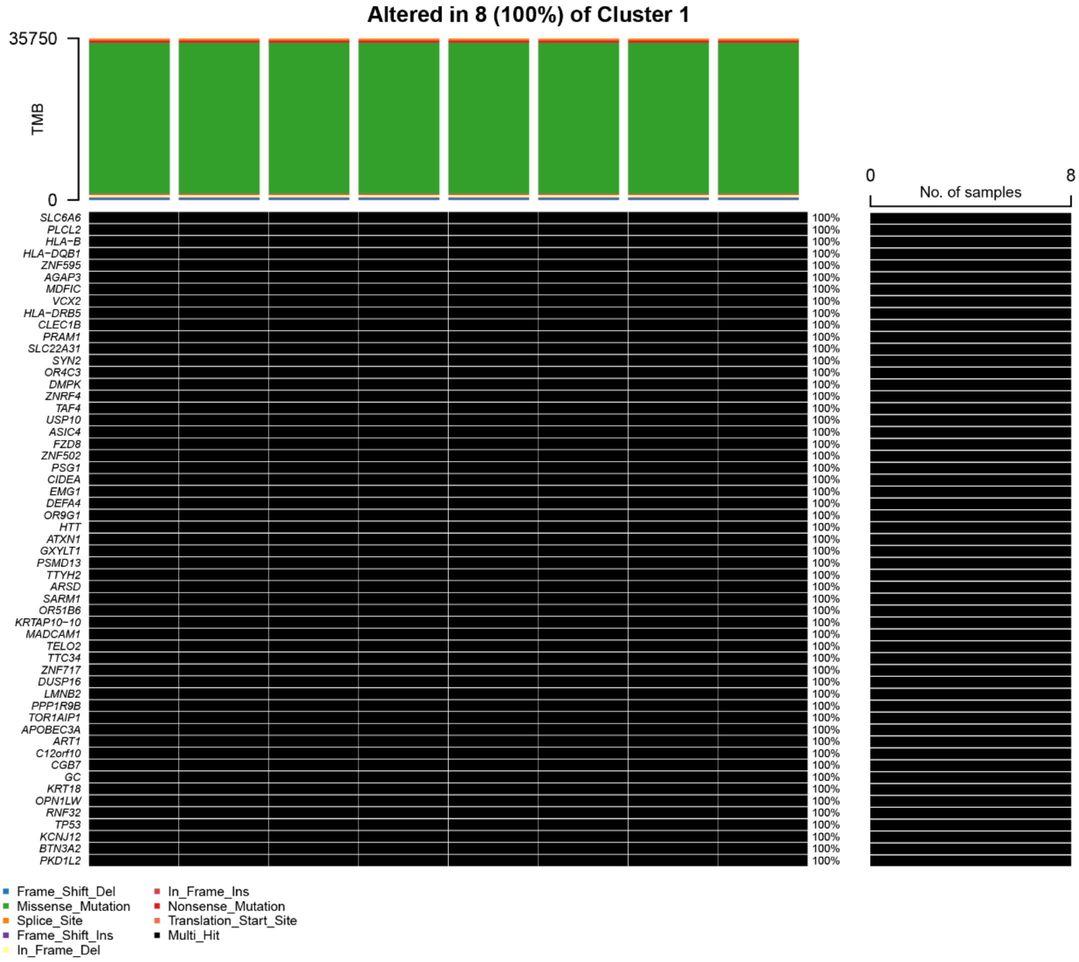

Figure S3. Distribution map of the 55 SMGs in cluster1, with multiple mutations occurring in all samples.

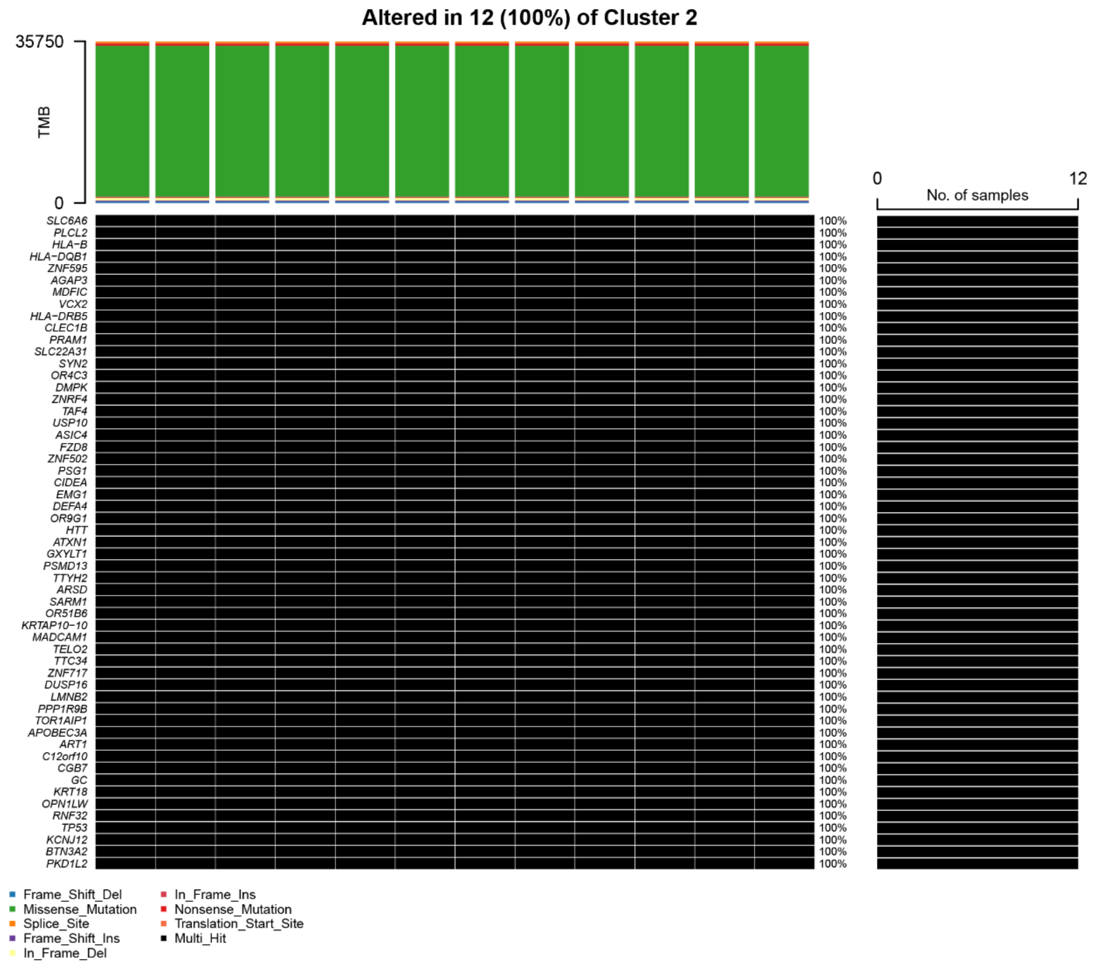

Figure S4. Distribution map of the 55 SMGs in cluster2, with multiple mutations occurring in all samples.

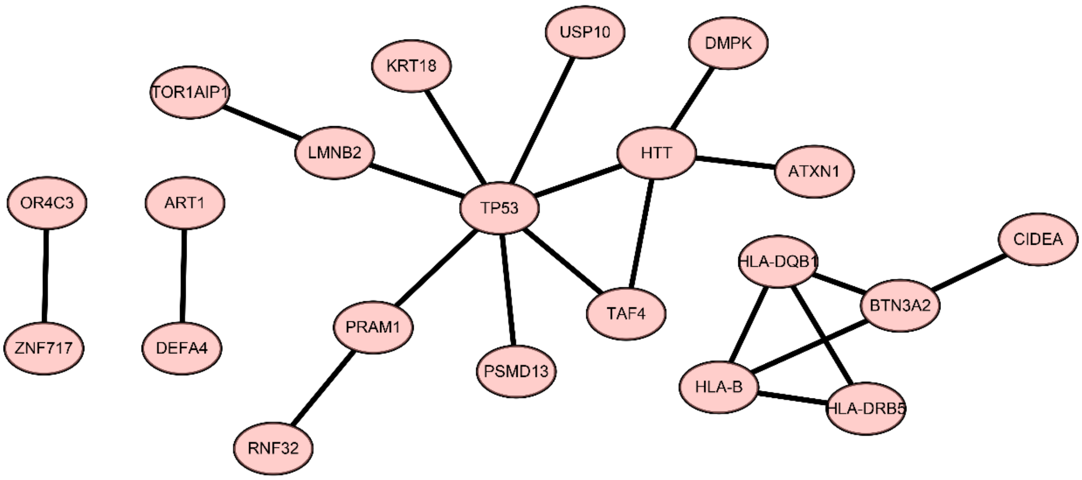

Figure S5. PPI networks of SMGs.
