# Supplementary figures and images for: Revealing the Potential Associations of Mutation-Related Genes with Lymph Node Metastasis in Gallbladder Cancer Through Transcriptome and Exome Sequencing
Source: Biomedicines. 2026 May 10;14(5):1076. doi: 10.3390/biomedicines14051076 (PMC13204380; doi:10.3390/biomedicines14051076)

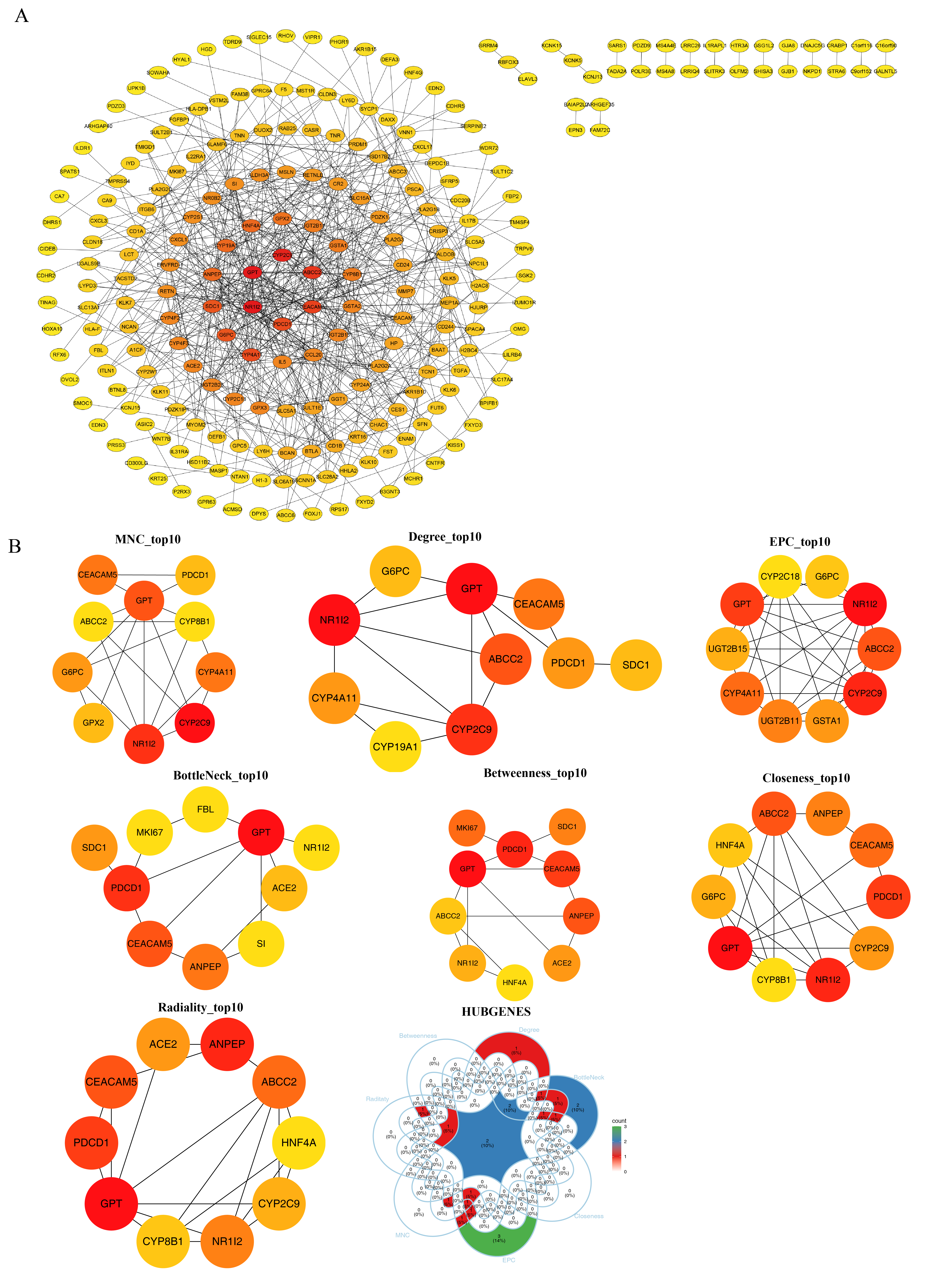

Supplement: Supplementary file 1 [file biomedicines-14-01076-s001.zip › Figure S1.tif]

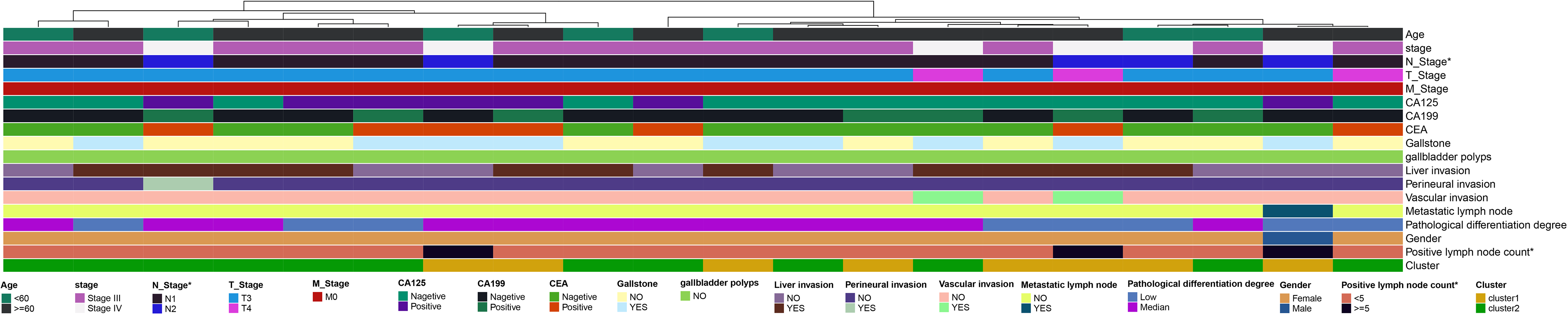

Supplement: Supplementary file 1 [file biomedicines-14-01076-s001.zip › Figure S2.tif]
